# Identifying Misbehaving Greedy Nodes in IoT Networks

**DOI:** 10.3390/s21155127

**Published:** 2021-07-29

**Authors:** Fatima Salma Sadek, Khaled Belkadi, Abdelhafid Abouaissa, Pascal Lorenz

**Affiliations:** 1 SIMPA Laboratory, USTO-MB Université des Sciences et de la Technologie d’Oran Mohamed Boudiaf, Oran 31000, Algeria; selma.sadek@univ-usto.dz (F.S.S.); khaled.belkadi@univ-usto.dz (K.B.); 2 IRIMAS/GRTC Laboratory, University of Haute Alsace, 68000 Colmar, France; abdelhafid.abouaissa@uha.fr

**Keywords:** security IoT, detection method, greedy nodes, DoS attack

## Abstract

One of the central communication infrastructures of the Internet of Things (IoT) is the IEEE 802.15.4 standard, which defines Low Rate Wireless Personal Area Networks (LR- WPAN). In order to share the medium fairly in a non-beacon-enabled mode, the standard uses Carrier Sense Multiple Access with Collision Avoidance (CSMA/CA). The nature of connected objects with respect to various resource constraints makes them vulnerable to cyber attacks. One of the most aggressive DoS attacks is the greedy behaviour attack which aims to deprive legitimate nodes to access to the communication medium. The greedy or selfish node may violate the proper use of the CSMA/CA protocol, by tampering its parameters, in order to take as much bandwidth as possible on the network, and then monopolize access to the medium by depriving legitimate nodes of communication. Based on the analysis of the difference between parameters of greedy and legitimate nodes, we propose a method based on the threshold mechanism to identify greedy nodes. The simulation results show that the proposed mechanism provides a detection efficiency of 99.5%.

## 1. Introduction

The Internet of Things is considered as an industrial revolution in the world of computing. Indeed, this technology interconnects a huge number of intelligent physical devices [1]. The intelligence of these devices lies in the fact that they are endowed with the capacity for learning and reasoning. In addition, they operate autonomously, since they do not require any human intervention [2]. The emergence of the Internet of Things has greatly contributed to the development of several fields. Its application extends from a simple smartphone, to smarthomes, and smartcities [1]. The use of WSN (Wireless Sensor Network) is also widely used in the context of IoT, since the sensors share data that they have previously detected, collected, and processed. For this purpose, the IoT uses the IEEE 802.15.4 communication infrastructure specifying the PHYsical (PHY) and Medium Access Control (MAC) layers in LR-WPAN (Law Rate Personal Area Network) [3]. Despite the many advantages offered by IoT, which have allowed to record progress in various fields, this technology remains vulnerable to cyber attacks. The IoT devices constraints in terms of resource restrictions (low memory, battery, computing and storage capacity, etc.) make them particularly vulnerable [4], and constitute another challenge when it comes to the development of optimized security tools and methods. The availability of network resources, and timely information, is one of the fundamental security requirements of the IoT [5], particularly in certain critical fields such as the smart medicine IoMT (Internet of Medical Things) [6], military field, or industrial field in which the availability of information and network resources in real time are essential, and where QoS (Quality of Service) is a major requirement. Neglecting such an important requirement in IoT networks can lead to very serious problems. For example, in a water dam in which sensors are deployed to supervise the water level, the closing or opening of dam valves and other sensitive actions, if a DoS (Denial of Service) or a selfishness attack occurs, the consequences are that the network will be saturated, and the sensors cannot transmit their collected data to the server. This can lead to a disaster, because this collected information is important for managing the water dam and it will not tolerate any delay in transmission. The DoS attack is considered the ultimate goal of the attackers. In order to achieve this goal, hackers use various methods. In IoT networks, there are several ways to carry out this type of attack. We cite, as an example, the sleep deprivation attack [4] and the jamming [7], flooding [4], or greedy behaviour attacks [8]. The first method is performed by an attacker who targets a specific sensor, which is the PAN coordinator most of time, because he has the most important role in the network. This attacker will deprive his target of sleep mode and force him to remain in reception mode, since he will send him a large number of messages [4], or ask him to perform an important calculation. This attack requires a powerful machine that has no resource constraints, which is not the case of sensors. The second type of attack is the jamming attack, which is an indoor attack performed by a sensor on the network. There are four types of jammer nodes. The first one is the constant jammer which continuously emits a radio signal on the transmit channel. The second is the deceptive jammer, which constantly injects packets into the channel. The third is the random jammer, which sends out packets in random time intervals [7]. Finally, the fourth is the reactive jammer which listens to the channel continuously and sends packets when the channel is detected busy, in other words, when another node is sending its packets [9]. The flooding attack uses one or various nodes to saturate the network, by sending regular messages to the target [4]. These attacks are interesting, but require a powerful battery, which is not the case of sensors. Finally, the last type of attack is the greedy behaviour attack, which uses a sensor called a greedy node or selfish node. This attack is both an aggressive and a smart DoS attack [8]. Its intelligence lies in the fact that the greedy node will saturate the network without being detectable, because it will impersonate a legitimate node. It will therefore not send packets to a specific target, nor randomly over the network. The greedy node will try to monopolize the transmission channel, while depriving other legitimate nodes of access to it [8]. To do this, the greedy node deliberately falsifies its CSMA-CA parameters in order to increase its chances of permanently accessing the transmission channel. It will therefore compete with the other legitimate nodes of the network for access to the channel, but it will have transmission priority most of time. Thus, a selfish node causes network saturation and degrades its quality of service by increasing collisions and lost packets, while impersonating a legitimate node. The advantage of this method is that a greedy node can sleep most of time which allows him to maintain an acceptable battery level compared to the saturation of the entire network executed by a single node.

Hence, the identification of a greedy node is essential to ensure the proper functioning of the IoT network. However, as it was mentioned above, the detection of greedy nodes is very difficult to implement in IoT networks. This is what motivated us to provide this work. Moreover, to the best of our knowledge, this study is the first to deal with this subject, and which effectively detects greedy nodes in an IoT network. To do this, we will start by executing the greedy behaviour attack by a malicious node. Then, we analyse the difference of parameter values of greedy and legitimate nodes. For each relevant parameter, a threshold is fixed, and any node deviating from those thresholds is considered as greedy node. In order to reduce the false positive and negative alarms, eight parameters were used, namely, packets sent, packets received, collision number, power, radio on rate, radio transmission rate, transmit power, and transmit duty cycle. The proposed method of detecting greedy nodes mainly boils down to three essential steps. The first one is collecting information and network traffic. The second one consists of analysing the collected information. Finally, the third step is the detection phase that is based on the threshold mechanism.

The paper is organized as follows. The Section 2 is related work. The Section 3 describes the IEEE 802.15.4 standard and the greedy behaviour attack. We present in the Section 4 the method used to identify the greedy node. Section 5 presents the simulations and the obtained results. Finally, we conclude the paper with a definite perspective in Section 6.

## 2. Related Work

While the greedy behaviour attack has been widely studied in IEEE 802.11 wireless networks, little work has been done in IEEE 802.15.4-based WSNs. Indeed, several techniques and methods for greedy node detection have been proposed in IEEE 802.11 wireless networks, including game-theoretic, fuzzy logic-based schemes; however, to the best of our knowledge, there is almost no work regarding greedy node detection and identification in IoT networks. We have selected the most recent works in the literature to present in this section. Works [10,11,12,13] focus on the characterization and modelling of the greedy behaviour attack.

The authors propose in [10] a model of greedy behaviour in wireless Ad Hoc networks. The greedy node increases its bandwidth by randomly choosing a backoff value within a range bounded by a misbehaviour threshold. This threshold represents a bandwidth limit that the greedy node must not exceed in order to avoid performance degradation. In order to prevent other nodes from sending their packets, the greedy node causes collisions with its neighbours’ frames except for the next-hop frames of its current flow.

In [11], the authors have studied the impact of greedy nodes in the IEEE802.11 WLAN network by combining timed automata and Discrete-Time Markov Chains (DTMC). The study proposes a formal approach for modelling CSMA-CA in IEEE802.11 networks containing greedy nodes. In order to monopolize the transmission channel, the greedy node modifies the Distributed Coordination Function (DCF) procedure. It does not execute the backoff procedure under any circumstances, due to the removing of the condition y = DIFS from the WaitingUntilTransmit and WirelessMediumSensing steps. Therefore, if the greedy node finds the channel free after carrying out a CCA, it immediately starts sending its packets without delaying the DIFS (DCF InterFrame Space). Likewise, if the channel has been detected busy, the greedy node does not execute a backoff and continues to sense the medium at each timeslot until it finds it free. If the packet from the greedy node collides with another, and transmission fails, the greedy node resumes its selfish behaviour by returning directly to the procedure WaitingUnitTransmit without observing any backoff. This study demonstrates that this behaviour negatively impacts the traffic of all nodes, including the compromised nodes themselves.

In [12], the authors propose a UPPAAL model of the greedy node in a WSN network using the IEEE 802.15.4e standard and star topology. Authors were particularly interested in the beacon-activated mode contention access period of slotted CSMA-CA. The proposed model consists of two automates, namely, Medium and Node. The first models the state of the wireless medium, while the second describes the behaviour of nodes transmitting frames during CAP. The greedy node is modelled by falsifying the following parameters: the length of the initial conflict window (CW0), the minimum value of BE (minBe), and the maximum value of BE (maxBe).

The authors propose in [13] the modelling of a greedy node in WSN networks based on unslotted IEEE 802.15.4 CSMA/CA, using the UPPAAL model-checker. The greedy node tries to monopolize the transmission channel by reducing its backoff, and by increasing its attempts to access the medium.

The authors of [14] consider the greedy behaviour attack in IEEE 802.15.4 CSMA-CA. They propose the use of a Markov chain model to demonstrate the impact of this attack on the network throughput performance degradation. For this purpose, the authors represent the CSMA-CA protocol as a discrete-time Markov chain. This study presents the results of the mathematical analysis of the CSMA-CA throughput with an exponential random backoff. In order to show the effects of the selective backoff attack on the network, two scenarios are possible. The first assumes that the greedy node sets its contention window parameter Cw to 1. In this case, it can occupy the channel at the first time slot without any waiting while the other devices wait to perform a CCA and detect that the channel is free before transmitting. In the second scenario Cw = 2, other devices in the nearby network can access the channel at the first time slot. This study only presents a mathematical analysis of the performance of the selfish node and the degradation of the throughput of other devices. However, there are no details on the methods for identifying greedy nodes within the IEEE 802.15.4 network. Moreover, the authors did not provide simulation parameters in terms of the number of packets sent or lost, the collision rate, or the battery level. These parameters are important to highlight in order to assess the difference in behaviour between legitimate and greedy nodes.

In [15], the authors propose a nonparametric statistical approach to detect malicious filtering nodes in mobile networks assisted by network coding. The proposed method is based on the Kruskal–Wallis statistical test. Honest users represent a single population, and all control packets generated by that population are examined. The proposed scheme is based on ranks, in order to detect whether different sets of samples belong to the same probability distribution. Concerning the modelling of the legitimate and egoistic nodes, the authors suppose that the nodes of the network produce in a periodic time interval ratios rm (t) ∈ {0,1}. A given node is considered legitimate if its ratio rm (t) = 1, and selfish if it is rm (t) = 0. Knowing that legitimate nodes can provide a false report with a probability called “pe”, due to channel errors, then those malicious nodes deliberately provide false reports with probability pf.

The works [16,17] are based on game of theory and fuzzy logic to detect greedy nodes and adopt a defence strategy in VANets networks. The authors propose a method combining the detection and identification of greedy nodes in VANets networks. This method is called GDVAN (Greedy Detection for VANETs). It uses linear regression to detect greedy behaviour attacks in the network and fuzzy logic to identify greedy nodes. Three metrics are used to identify selfish nodes, namely: number of connection attempts, node connection time, and average wait time between connections.

In [18], the authors propose a countermeasure to misbehaviour attacks and optimized energy consumption. They propose a model of the IEEE 802.15.4 standard in the form of a dynamic game, where the different nodes of the network represent players able to assess the state of the game, then select and adapt their game strategies while optimizing energy consumption. Concretely, the dynamic MAC model proposed works exactly as the IEEE 802.15.4 standard in the normal state, but when the state of the game changes, the nodes individually change their game strategies by modifying their contention parameters to improve their energy consumption. It should be noted that the state of the game is evaluated only by two main parameters: the number of transmission failures, and the failure to execute the CCA (Clear Channel Assessment). Although the authors assure by the simulation results that this proposal allows the CSMA-CA and GTS mechanisms to be resilient to greedy behaviours in IoT networks, this method is likely to work badly in the case where a large number of nodes are saturated. Furthermore, this study does not in any way perform the greedy behaviour attack by a malicious node in order to demonstrate its impact on the WSN network before proposing this countermeasure. The simulation was performed in a probabilistic wireless network simulator based on MATLAB. The authors simulated four nodes, playing the native IEEE 802.15.4, while four other nodes are playing the proposed dynamic game. However, the proposed countermeasure solution was achieved without the presence of a greedy node. Therefore, this proposal may not work well when the IEEE 802.15.4 network is attacked by a misbehaving greedy node. In addition, this proposal does not take into account the reality of the variation in the level of fees, and no system for detecting greedy nodes has been implemented.

The authors propose in [19] the cooperative detection of the nodes of misbehaviour, which falsify the size of the contention window in a CSMA-CA protocol with 802.11 slots. To this end, two strategies are proposed. The first aims to observe network traffic in order to detect if there is an anomaly. To do this, each node observes its neighbour for a time interval using ACK packets. The second strategy is reaction, where the legitimate nodes have the choice to accept the selfish behaviour of the malicious nodes, or to retaliate by adopting the same behaviour in order to be able in turn to gain access to the transmission medium.

Works [8,20] use Petri net time to suspect the presence of greedy nodes. The authors of [20] based their approach on that of [13] to characterize the greedy node, then proceeded to model the normal and greedy node using temporal Petri nets. Based on the numerical results of the simulation performed on the TINA platform, the authors state that the increase in the number of lost packets and the collision rate in a WSN network are the two main parameters to take into account to suspect the presence of greedy nodes. Therefore, they conclude that a node is suspected of being greedy if it has a high successful packet transmission rate and a low lost packet rate compared to the rest of the nodes in the network. The authors suggest that the suspected greedy node should be monitored to confirm the verdict.

In [8], the authors are particularly interested in the energy consumption of legitimate nodes in the presence of greedy nodes in a Zigbee network based on IEEE 802.15.4 without slot. They simulate different scenarios using time-based Petri nets. In the section on detection, the authors state that it is very difficult to detect the greedy behaviour attack because most of the time it can be mistaken for a transient overload of the network, especially since a given node cannot know the number of nodes trying to access the medium in a given period of time, and this is what leads to the increase in false suspicions. The authors did not proceed to the detection of malicious greedy nodes; they recommend to reserve this task for certain critical areas where QoS and service availability are prior requirements. They therefore propose a simplified system allowing to suspect a greedy behaviour attack but without identifying the greedy nodes. This task is granted to the PAN coordinator who will monitor the network parameters within a given monitoring period. The network is suspected of being attacked if the percentage of failures is greater than 60%, while the Failure/Collision ratio is less than 5. If this behaviour persists throughout the monitoring period, the PAN informs the nodes of the network so that they perform a countermeasure allowing them to manage their energy consumption reasonably.

Based on the related study presented in this part, we affirm that there is no study that addresses the detection and identification of greedy nodes present in an IoT network based on IEEE 802.15.4. This is what motivated us to tackle this subject and to propose this accurate and efficient detection method.

## 3. Selfish Behaviour in IEEE 802.15.4

### 3.1. IEEE 802.15.4 Characteristics

The IEEE 802.15.4 standard defines the Physical Layer (PHY) and Medium Access Control (MAC) specifications for Low Rate-Wireless Personal Area Networks (LR-WPAN) [3].

This standard is responsible for managing communications that are limited in terms of cost, power consumption, and speed. It is associated in the majority of implementations with the use of ZigBee [21] and 6LoWPAN protocols in the ISM 2.4 GHz band [3]. It mainly uses two physical devices: FFD (Full Function Device) and RFD (Reduce Function Device). A FFD can communicate with any device on the network, while a RFD can only communicate with a FFD [3]. Two topologies are supported by the IEEE 802.15.4 standard: the star topology, and the peer-to-peer topology. Cluster tree topology is considered as a special case of peer-to-peer topology imposing hierarchical topological specifications on network formation, and using more FFDs than RFDs. The star topology is a centralized topology where communications are carried out only between the PAN coordinator and the nodes within its radio range. This topology uses the beacon enable mode using slotted CSMA/CA; it is a so-called synchronized mode, which uses a superframe whose role is to synchronize the nodes with the coordinator [3].

In the peer-to-peer topology, communications are bidirectional between nodes, and without any restriction, since the intermediate nodes manage to relay the packets to the destination. This topology is more advantageous in terms of flexibility and scalability of the WSN network. The peer-to-peer topology uses the non-beacon enable mode. It is an asynchronous mode based on periodic wake-ups (duty cycle) of the nodes either to send or to check if they have messages which are intended for them. The channel access mechanism used in this mode is unslotted CSMA/CA [3]. We consider in our study a peer-to-peer topology of a wireless sensor network.

### 3.2. Greedy Behaviour Algorithm

Before explaining the operation of the greedy node, it is necessary to present the algorithm of the unslotted CSMA/CA protocol, since the greedy node falsifies several parameters of the protocol in order to gain bandwidth. The main parameters used in unslotted CASMA/CA are:

- Backoff Exponent (BE): It is a value used in the calculation of the backoff delay.
- Clear Channel Assessment (CCA): The CCA is performed by each node in the network in order to check if the channel is free before transmitting. The CCA duration is equal to 8 symbols.
- Number of Backoff (NB): It is a counter of the number of failed channel access attempts.
- MacMinBE: It is the minimum value of BE. This value varies between 0 and 3. By default, CSMA/CA uses 3.
- MacMaxBE: It is the maximum value of BE. It is equal to 5.
- MacMaxCSMABackoffs: It is the maximum number of attempts to access the channel.
- UnitBackoffPeriod: It is equal to 20 symbols by default.

In order to ensure the equitable sharing of the medium, unslotted CSMA/CA carries out the following steps. The first step is to initialize the parameters NB to 0, and BE to MacMinBE. Then, a backoff period is calculated in the second step in order to avoid collisions. The algorithm randomly chooses a value in the interval (0, $\left(2^{\mathrm{BE}}\;-\;1\right)$). This value is multiplied by UnitBackoffPeriod to obtain the duration of the backoff.

The third step of the algorithm is the CCA procedure. If after the execution of a CCA the channel is free, the node starts sending its packets by executing the fifth step of the CSMA/CA algorithm. Otherwise, it is the fourth step which is executed and which consists in incrementing NB by 1, and assigning to BE the minimum value between BE+1 and MacMaxBE. If NB has reached its maximum value MacMaxCSMABackoffs, the packet is dropped, and an error message is transmitted to the upper layer. Otherwise, go to step 2, i.e., recalculate the backoff period.

The fifth step is the transmission of the packets, after having checked that the channel is free by the CCA procedure. Note that collisions can occur at this stage if two or more nodes are transmitting at the same time.

The ultimate goal of the greedy node is to monopolize the transmission channel in order to deprive other legitimate nodes from transmitting their packets. For this, the greedy node tries to increase its chances of accessing the channel compared to other legitimate nodes of the network, and doing this by reducing both its backoff period and the duration of the CCA, and by increasing its attempts to access to the medium. For this purpose, the UnitBackoffPeriod parameter is divided by 4. It will therefore be equal to only 5 symbols instead of 20. Then, the two parameters, MacMinBE and MacMaxBE, will be respectively equal to 0 and 1. Therefore, the value of BE will vary between 0 and 1. Hence, the maximum duration of the backoff executed by the greedy node will be 80 us (5 symbols). The duration of the CCA is also divided by 4, and will therefore be equal to 2 symbols. Finally, the number of channel access attempts is doubled by doubling the MacMaxCSMSBackoff value which will be equal to 10 instead of 5.

**Figure 1:**
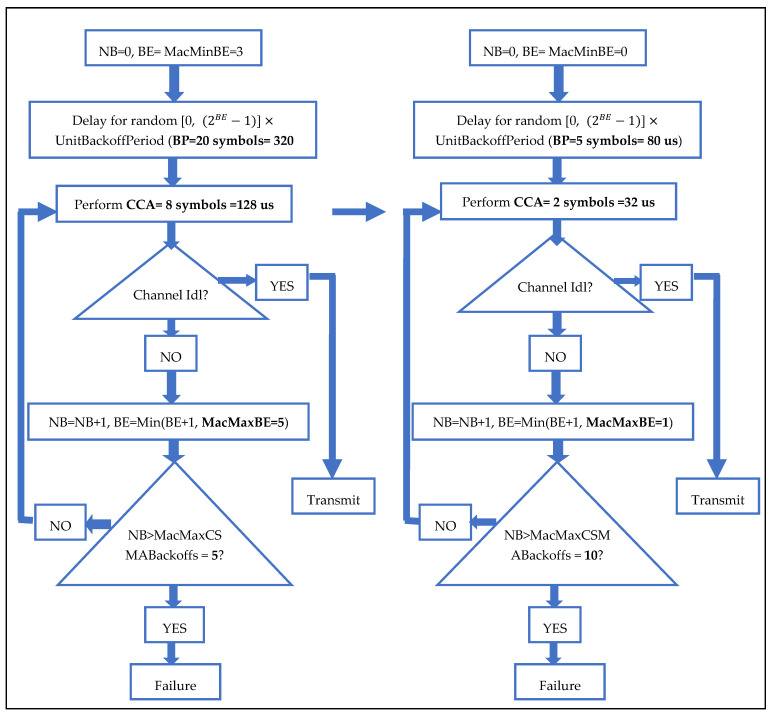
The difference between CSMA/CA algorithm and greedy node algorithm.

The Figure 1 demonstrates the difference between the CSMA/CA algorithm of a legitimate and greedy node.

## 4. Detection of Selfish Nodes

As mentioned in the introduction, we have chosen to analyse the most relevant parameters, i.e., those influenced by the behaviour of the greedy node. The number of packets sent parameter is one of the most relevant parameters since the greedy node sends more packets than a normal node. Next, we analyse the number of packets received by each node, because if the greedy node monopolizes the channel partially or totally, it will prevent other legitimate nodes from sending their packets. As a result, it will receive fewer packets than the others. The number of collisions caused by each node is also a parameter to be considered, since the greedy node falsifies its listening channel parameters (number and duration of CCA). It will therefore observe less time to check the channel. Hence, it will cause more collisions. The radio status settings are indicative of greedy behaviour, since they inform about the radio transmission rate (radio Tx) and the duration when the radio is on to either send or receive. Finally, the energy consumption parameters (power, transmit power) and transmit duty cycle are also important to be considered since the greedy node consumes more energy to send its packets, and performs more wake-up cycles than the other nodes.

In order to determine if a node behaves normally or if it adopts a greedy behaviour, we use the eight parameters previously cited. The use of those eight parameters offers an efficient and accurate detection solution, because it allows for reducing the false alarms since a given node should satisfy eight different conditions specific to each parameter, for being determined a greedy node by our algorithm. For this purpose, we set thresholds for each parameter.

For the parameters that tend to increase in the case of greedy behaviour, such as packets sent, collision, transmit power, transmit duty cycle, power, radio on, and radio Tx, we set a maximum threshold for them. All values that are lower than those thresholds are considered “normal”, while values that exceed them are considered abnormal. Regarding the packets received parameter, it is the only parameter that undergoes degradation during greedy behaviour. Therefore, we set it a minimum threshold, and all values lower than this threshold are considered abnormal. We propose the use of a statistical method based on the mean and standard deviation to determine the minimum and maximum thresholds.

Thus, the minimum and maximum thresholds for each P parameter are calculated as follows:

(1) $$Th_{max}\left(P\right)\;=\;M_{P}\;+\;\alpha .\sigma_{P}\;=\;\frac{\sum_{k=1}^{n}P}{N}\;+\;\alpha .\sigma_{P}$$

(2) $$Th_{min}\left(P\right)\;={\;M}_{P\;}-\;\alpha .\sigma_{P\;}=\frac{\sum_{k=1}^{n}P}{N}-\alpha .\sigma_{P}$$

where $M_{P}$ represents the mean of P, $\sigma_{P}$ the standard deviation of P, and α is an adjustment variable that varies depending on the parameter used. N is the total number of sender nodes in the network, and n is the last sender node.

For each parameter, the α value of the maximum threshold is calculated as follows:

(3) $$L\;<\;\;Th_{max}(P)\;<\;G$$

where G is the value of the greedy node for a given parameter, and L is the upper value of a legitimate node for the same parameter. Note that L has to be lower than G. If this condition is not true, we take the next upper value of a legitimate node.

By replacing $Th_{max}$ by its formula, we obtain:

(4) $$\frac{L-\frac{\sum_{k=1}^{n}P}{N}}{\sigma_{P}}\;<\;\alpha \;<\;\frac{G-\frac{\sum_{k=1}^{n}P}{N}}{\sigma_{P}}$$

The α value of the minimum threshold used for the packets received parameters calculated as follows:

(5) $$G\prime \;<\;Th_{min}(P)\;<\;L\prime$$

where G′ is the packets received number of greedy node, and L′ is the lower value of packets received by a legitimate node.

By replacing $Th_{min}$ by its formula, we obtain:

(6) $$\frac{\frac{\sum_{k=1}^{n}P}{N}-\;L\prime \;}{\sigma_{P}}\;<\;\alpha \;<\;\frac{\frac{\sum_{k=1}^{n}P}{N}-\;G\prime }{\sigma_{P}}$$

The detection of greedy nodes requires several steps. The first consists of the collection of network information from the sink node, and store it in a database.

**Figure 2:**
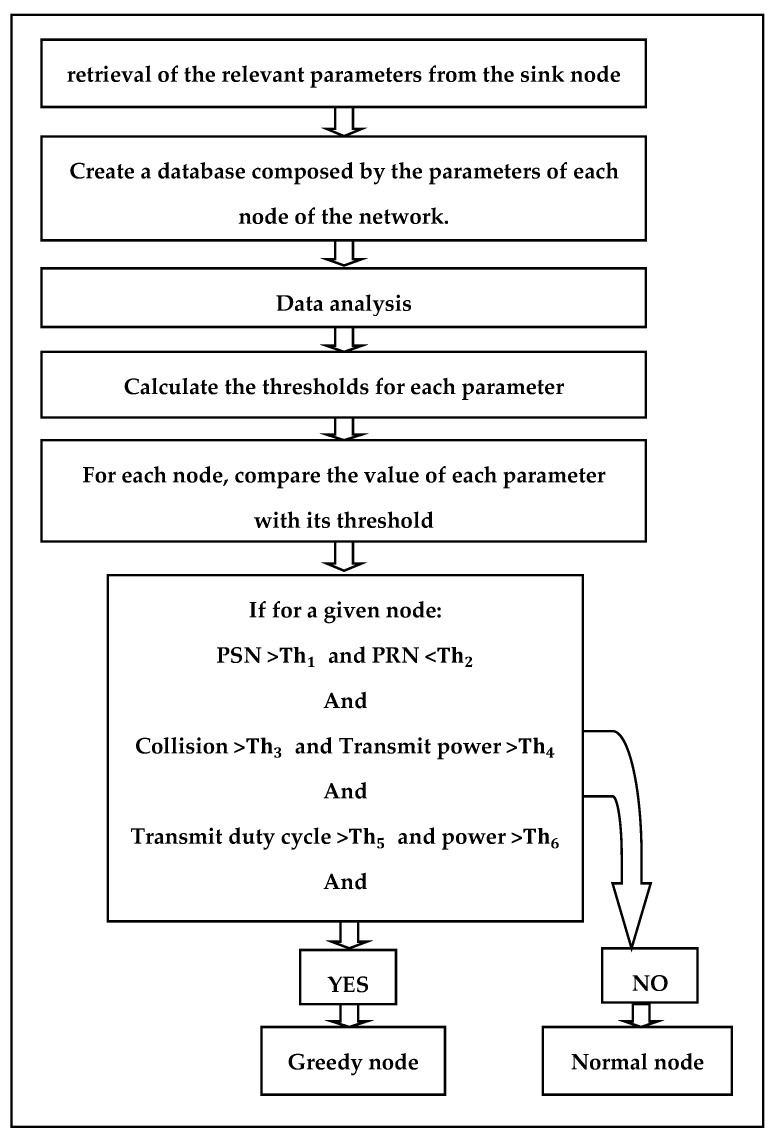
Greedy node algorithm identification steps.

The next step is the data analysis. This step is important to understand the impact of the greedy node on each of the parameters. On the basis of this analysis, the min/max thresholds are determined and calculated for each parameter according to its variation. As presented in the Figure 2, the test step is carried out by comparing, for each node, the values of each parameter with their appropriate thresholds.

Finally, the last step is to determine if a node is legitimate or greedy. A given node is considered greedy if its eight parameters belong to the so-called abnormal values (i.e., greater than or less than the thresholds). In other words, the greedy node must have sent more packets than the threshold ${\mathrm{Th}}_{1}$, and the number of collisions caused by the greedy node must exceed the threshold ${\mathrm{Th}}_{2}$, and the number of packets received by this node must be less than ${\mathrm{Th}}_{3}$, and the Transmit power, Transmit duty cycle, Power, Radio on (%), and Radio Tx (%) parameters must be respectively upper than their appropriate thresholds: ${\mathrm{Th}}_{4}$, ${\mathrm{Th}}_{5}$, ${\mathrm{Th}}_{6}$, ${\mathrm{Th}}_{7}$, and ${\mathrm{Th}}_{8}$.

## 5. Simulation and Results

In order to analyse the behaviour of a greedy node in a WSN network based on IEEE 802.15.4, we consider the following scenario: We simulate a scalable network composed of 5, 10, 15, and 20 nodes sender, and a sink node responsible for collecting network information. We inject in each topology one greedy node.

We have chosen to execute the attack on the network using a single greedy node, because the use of several malicious nodes will create competition between them for access to the medium since they use the same CSMA/CA parameters. This will lead to a degradation of their performance, while the aim of this study is to analyse the behaviour of the selfish node and its influence on network parameters. This analysis will be used to calculate the thresholds, allowing for distinguishing the normal values from the abnormal ones. In addition, the use of a single greedy node on a scalable network has demonstrated its performance and effectiveness since it has managed to create a significant disturbance in a network made up of a considerable number of legitimate nodes.

It should be noted that each simulation was repeated two times. Thus, the experimental study is done on 16 distinct simulations. The simulations composed of 5, 10, 15, and 20 will be called in the rest of the article $N_{1},N_{2}$, $N_{3}$, and $N_{4}$ respectively. Given that each simulation has been performed twice, $N_{i.2}$ represents the second simulation for each i network. We chose to use the Cooja simulator of the Contiki OS [22], to carry out the different simulations, and since it requires fewer resources to run [23], it is therefore the most appropriate for an IoT environment known for its very limited resources. The topology used in each simulated network is peer to peer, and the nodes are randomly positioned on a square grid, their coordinate being picked randomly according to uniform random variables. It should be noted that the surface of the grids vary according to the density of each network.

The wake-up frequency used in the ContikiMAC primitive is 8 Hz. The duration of each simulation is 30 min (according to the simulator time). The transport protocol used is UDP, and the routing protocol is RPL. The tow files updated to falsify the CSMA/CA parameters are csma.c in the MAC layer, and contikimac.c in the RDC (Radio Duty Cycle) layer of contiki OS.

We collect network traffic from the sink node to build a database composed of the most relevant network parameters mentioned above. The sink node receives every 60 s a report from each node of the network containing all information and parameters values that concern them such as power, transmit power, transmit duty cycle, CPU power, churn, listen power, etc. The sink node collects this information in a database and updates it each time it receives a report from one of the nodes in the network. Regarding the number of packets sent and received, and the number of collisions for each node, we calculated them with a script that we implemented which receives the contiki OS timeline file as input.

It should be noted that we did not take into account the values of the sink node neither in the analysis of the results nor in the calculation of the thresholds, since the sink node does not have the same characteristics as sender nodes as it periodically collects network data.

### 5.1. Greedy Behaviour Analysis Results

The step of analysing network traffic in the presence of the greedy node is essential for the detection, because it allows for understanding the impact and the degree of influence of the greedy node on each parameter. The analysis of the behaviour of the selfish node allows not only fixing the thresholds, but also determining the criteria allowing for distinguishing the greedy node from the legitimate ones.

In order to summarize the results, we calculated the average of each parameter for the set of legitimate nodes. Below are the simulation results.

**Figure 3:**
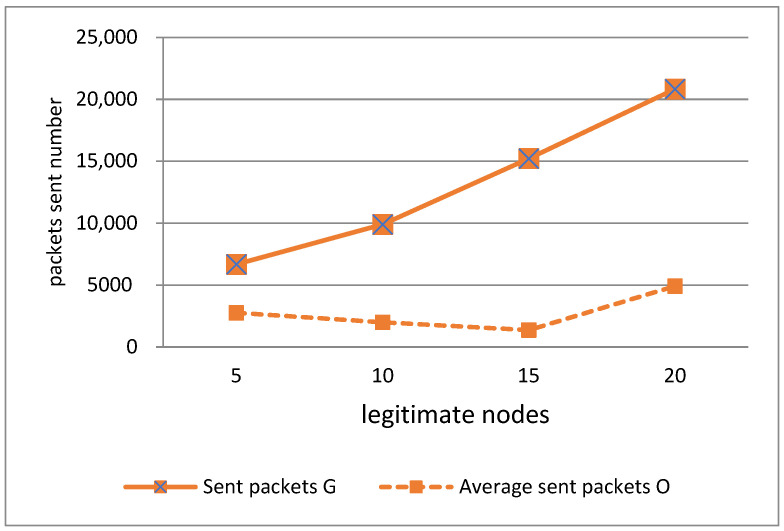
Packets sent by legitimate and greedy nodes.

In Figure 3, sent packets G represents the number of packets sent by the greedy node, and sent packets O represents the number of packets sent by the other nodes of the network. This figure clearly shows that in each of the networks, the number of packets sent by the greedy node is significantly higher than the number of packets sent by all the other nodes of the network, which demonstrates that the greedy node clearly monopolizes the transmission channel especially in networks 10, 15, and 20, where the number of legitimate nodes is high, and the competition for channel access is increased. We note that the difference between the number of packets sent by a single greedy node and all the nodes of the network is significant. This situation generates a denial of service because the legitimate nodes of the network do not manage to access the transmission channel, which deprives them of sending their packets. As explained in the previous section, legitimate nodes have only five attempts to access the channel before dropping their packets, and each time a CCA is executed to check if the channel is free to transmit, they find the channel occupied by the greedy node. Once the five channel access attempts are exhausted, they are forced to drop their packets, and will therefore be in a denial of service situation, which is dangerous if one of the nodes wants to transmit important information.

According to Figure 3, the number of packets sent is an important parameter to consider in the detection algorithm of the greedy node, since it clearly makes it possible to distinguish between a legitimate node and a greedy node, because the latter sends more packets than all the legitimate nodes of the network.

**Figure 4:**
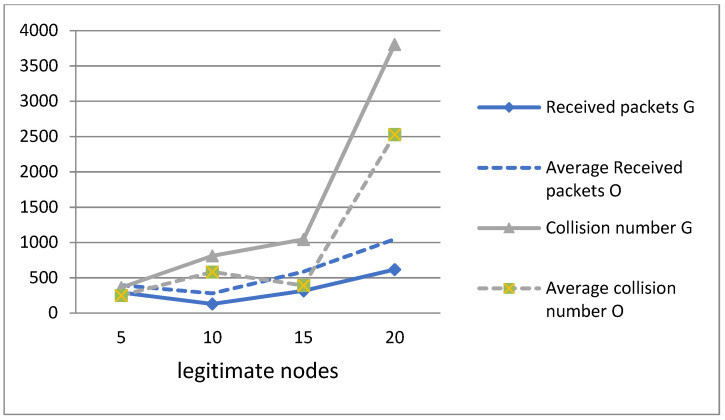
Received packets and collision number of legitimate and greedy nodes.

In Figure 4, the G represents the greedy node, and the O represents the other nodes in the network. We notice in this figure that the number of collisions caused by the greedy node is a little higher than the other nodes. This is explained by the fact that the greedy node executes a CCA of only two symbols, which is less than eight symbols for a legitimate node, and also less than the duration of the RTT (Round-trip time) which is the duration, from when a sender node sends a request to when it receives a response from a receiver node. Therefore, the greedy node does not have enough time to ensure that the transmission channel is free before starting to send its packets. Although the greedy node performs a CCA of only two symbols, its collision rate is not significantly high compared to other nodes, as this situation rarely occurs due to the random calculation of the backoff. Thus, by dividing the CCA by 4, i.e., by reducing it to two symbols instead of eight for a legitimate node, the greedy node increases its probability of accessing the medium and prevents other nodes from transmitting their packets. Therefore, the number of packets sent by a greedy node is significantly high (see Figure 3) compared to other nodes, while the number of collisions is slightly higher.

Regarding the number of packets received, as expected, the results of the simulations presented in Figure 4 demonstrate that the greedy node receives fewer packets than the other nodes, since it monopolizes the transmission channel and creates a denial of service in the network by depriving other nodes of access to it.

In short, we note in Figure 4 that during greedy behaviour, the number of collisions increases slightly, while the number of packets received decreases. As a result, these two parameters allow our detection algorithm to gain in accuracy.

**Figure 5:**
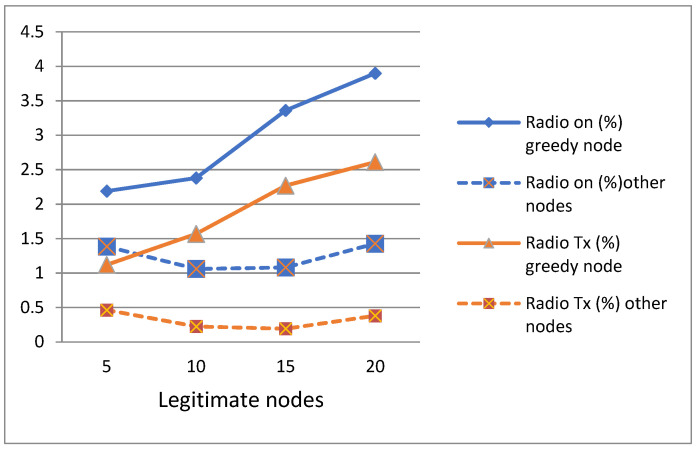
Radio on and radio Tx of greedy and legitimate nodes.

In Figure 5, we have represented the two states of the radio (on, and in transmission (Tx)) of the greedy node and the other nodes of the network. Both parameters are presented in percentage. We find that in each network, the greedy node keeps its radio on longer, and performs more radio transmission than other legitimate nodes, which is normal because it sent more than the other nodes of the network. According to Figure 5, this difference in value of the two parameters is very significant between the greedy node and the legitimate nodes; this is why we have added these two parameters in the detection algorithm.

**Figure 6:**
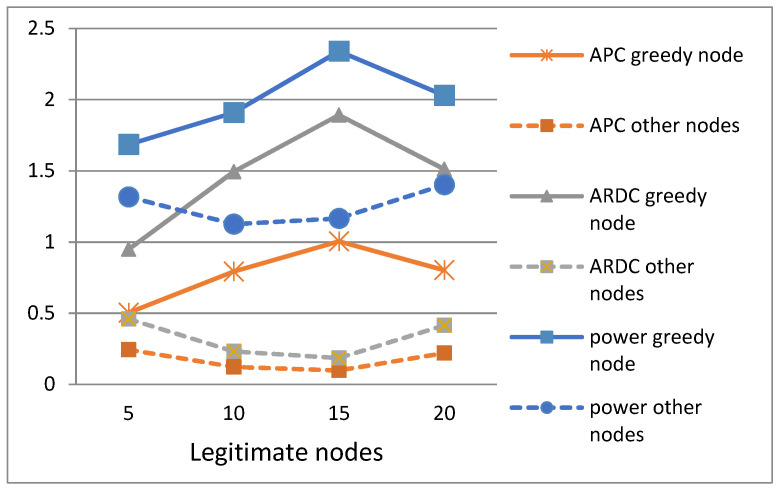
Power, APC, and ARDC of greedy and legitimate nodes.

In Figure 6, APC represents Average Power Consumption in transmitting state (transmit power). ARDC represents the Average Radio Duty Cycle in transmitting state (transmit duty cycle). This latter parameter informs about the wake-up frequencies performed by the node to transmit its packets. We notice in this figure that the three parameters APC, ARDC, and power of the greedy node are clearly superior than the other legitimate nodes. These three parameters indicate that the greedy node consumes more energy because it carries out more transmission than the rest of the nodes, knowing well that a device in transmission or reception mode consumes five times more energy than in any other state [24].

According to Figure 6, these three parameters are indicative of a greedy behaviour, since the energy consumption increases drastically at the selfish node compared to the rest of the nodes of the network. For this reason, these three parameters were also chosen as metrics to increase the efficiency of the detection algorithm. We observe that as the number of parameters that identifies greedy behaviour increases, the detection of greedy nodes will be more efficient in terms of effective detection and reduction in false alarms. Thus, with our mechanism, it is now very difficult to confuse a legitimate node with a greedy one. Furthermore, for a false alarm to occur, the legitimate node must be able to satisfy all eight conditions of greedy behaviour, which is very difficult to achieve.

Based on the analysis of the parameters previously carried out, we conclude that the parameters, number of packets sent, radio transmission rate (radio Tx), wake-up frequency rate (radio on, ARDC), as well as the power consumption (power, APC) of the greedy node are significantly higher compared to those of other legitimate nodes in the network. Concerning the level of the parameter, number of packets, received by the greedy node, it is low compared to the legitimate nodes. Finally, the greedy node causes a bit more collision than the rest of the nodes. The analysis results of the greedy node parameters are summarized in Table 1.

### 5.2. Thresholds Setting

The identification of the greedy node present in the IoT network represents the ultimate goal of this study. Based on the result of the network parameters analysis of the selfish node summarized in Table 1, we propose the use of a statistical method based on the mean and the standard deviation, as well as an adjustment value α to determine the minimum and maximum thresholds. For each of the eight thresholds, the α value must be calculated. For the parameter number of packets sent, the calculating process of the α value is as follows:

According to Table 2 representing the network parameter database of $N_{4}$, the number of packets sent by the greedy node (G) is 20,832 packets, while the upper number of packets sent by a legitimate sender node ($S_{i})\;$is 16,426 packets. Since 16,426 is lower than 20,832, L = 16,426.

Hence, according to Equation (3):

(7) $$L\;<\;Th_{max}\;(\mathrm{Packets}\;\mathrm{sent}\;\mathrm{number})\;<\;G$$

According to Equation (4):

(8) $$\frac{20832-5671.190476}{4502.051461}\;<\;\alpha \;<\;\frac{16426-5671.190476\;}{4502.051461}\;\left(2.38\;\leq \;\alpha \;\leq \;3.37\right)$$

The same method of calculating of α intervals for the threshold “number of packets sent” parameter was carried out for each of the remaining 15 simulations. The results are summarized in Table 3.

In order to maximize the efficient detection of greedy nodes, the upper bound of the α interval should be the lower value among the 16 intervals. Then, the reduction in false alarms is done by maximizing the lower bound. We therefore take the upper value among the 16 intervals provided that this value is lower than the value chosen for the upper limit.

Thus, we obtain:

1.5 ≤ α ≤ 1.75(9)

According to the experimental study carried out on the 16 simulations, the best result is obtained with α = 1.75.

By replacing the obtained value of α in (1), the equation of the threshold appropriate to the parameter number of packets sent is:

(10) $$Th_{max}\;(\mathrm{Packets}\;\mathrm{sent})\;=\;M_{Packets\;sent}\;+\;1.75\;\times \;\sigma_{Packets\;sent}$$

The same method of calculation was applied to determine the eight thresholds appropriate for each of the parameters. The results are shown in Table 4.

### 5.3. Greedy Node Identification Results

Finally, the last step consists of testing the proposed detection method on 16 different simulations. For each simulation, the parameter of each node is compared with its threshold. A node is identified as greedy if its eight parameters are outside the set thresholds. If only one of these parameters is within the so-called normal values, the node in question will be considered as legitimate.

The proposed greedy node detection algorithm receives as input the database of all nodes in the network with their parameters as indicated in Table 2. It will then calculate the eight thresholds appropriate for each parameter. In order to detect greedy nodes in the network, the algorithm proceeds by elimination. It starts with the first parameter, namely the number of packets sent. It will compare the number of packets sent from each node of the network with the threshold ${\mathrm{Th}}_{1},$ it will therefore browse the database table starting with the first node, until the last. If the number of packets sent from a given node is less than the threshold ${\mathrm{Th}}_{1}$, the node is considered legitimate. Otherwise, it tests its second parameter, and so on. If a given node satisfies all conditions, i.e., its eight parameters are considered abnormal, the node in question is determined to be greedy. It is enough that a single parameter is normal; the node will be determined as legitimate.

**Figure 7:**
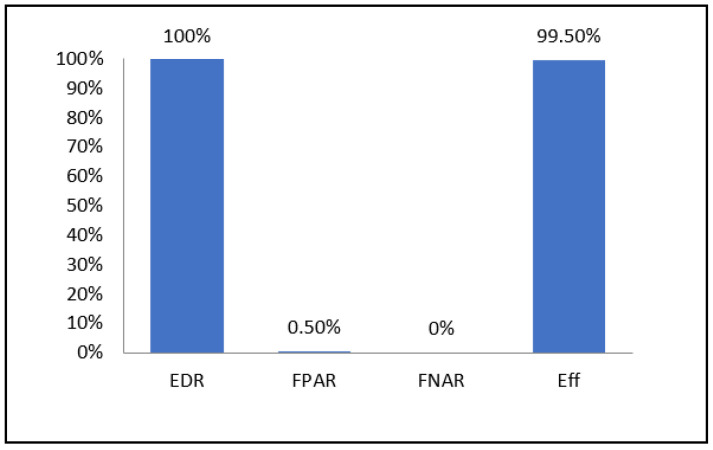
Greedy node identification results.

The proposed method was applied on 200 legitimate nodes and 8 greedy nodes. The results of these tests are presented in Figure 7 which demonstrates the detection efficiency based on the following four metrics:

1. Effective Detection Rate (EDR): It is the ratio between the number of nodes detected greedy by the proposed method and the total number of greedy nodes.
2. False Positive Alarm Rate (FPAR): It is the ratio between the total number of legitimate nodes in the 16 simulations and the number of legitimate nodes detected greedy by the proposed method.
3. False Negative Alarm Rate (FNAR): It is the ratio between the total number of greedynodes in the eight simulations and the number of greedy nodes identified as legitimate nodes.
4. Efficiency (Eff) represents the efficiency of the detection algorithm and which is calculated as follows:
  Eff = EDR − (FPAR + FNAR)(11)

The simulation results demonstrated an efficiency of 99.5%. The effective detection rate is 100% since the proposed method has allowed the identification of all greedy nodes in the eight compromised networks. Therefore, there was no false negative alarm. On the other hand, there was only one false positive alarm in network $N_{3}$. Thus, 1 in 200 legitimate nodes has been detected as being greedy.

In order to ensure the efficiency of our algorithm, we simulated two new networks by increasing the number of legitimate nodes, and reducing the simulation time to 10 min. The goal is to check if the algorithm detects the greedy nodes in topologies which have not been taken into account in the calculation of the thresholds. For that we simulated two networks $N_{5}$ and $N_{6}$ composed respectively of 25 and 30 legitimate nodes, and a single greedy node. In both simulations, greedy nodes were detected, and no false alarms were recorded. Table 5 represents the database of the N_6_ network.

Table 5 shows that although the nodes S1 and S2 send more packets than the greedy node, they were not considered by our algorithm to be a selfish node. Although the parameter number of packets sent and collision rate of the two nodes exceeds their appropriate thresholds, the number of packets received by S1 and S2 is respectively equal to 8229 and 2582, while the threshold has been set at 460.387298, knowing that the values considered as abnormal must be below this threshold. This condition is sufficient for considered them as legitimate nodes. The malicious node is the only one that was considered to be greedy since it is the only one that meets all the criteria. In short, on 55 legitimate nodes there was no false alarm, and in both networks the algorithm correctly detects the greedy node. This proves the efficiency and the precision of the proposed algorithm.

## 6. Conclusions

Detecting the greedy behaviour attack in IoT networks is important, but identifying malicious misbehaving nodes is essential to isolate them from the network. The main contribution of this paper is the proposal of a method for identifying greedy nodes in WSN networks based on IEEE 802.15.4. The proposed method consists of the analysis of eight parameters considered relevant for this type of attack. Based on the analysis results obtained, we assign to each of the parameters an appropriate threshold calculated from the mean, the standard deviation, and an adjustment value. A node is considered as greedy if, and only if, its eight parameters are outside of the set thresholds. The work presented in this paper is the first that deals with the detection of greedy nodes in IoT networks. The proposed method has demonstrated a detection efficiency of 99.5%. The perspectives of this work consist in the proposal of a method allowing for isolating the greedy nodes which have been identified in order to ensure the fairness in the sharing of the medium.

## Figures and Tables

**Table 1:** Evaluation of greedy node parameters level.

Parameters	Evaluation
Packets sent	Very high
Packets received	Low
Collision	High
Transmit power	Very high
Transmit duty cycle	Very high
Power	Very high
Radio on (%)	Very high
Radio Tx (%)	Very high

**Table 2:** The compromised $N_{4}$ settings database.

Node	Packets Sent	Collision	Packets Received	Transmit Power	Transmit Duty Cycle	Power	Radio on (%)	Radio Tx (%)
S 1	16,426	8439	4933	0.2	0.376	1.601	1.66	0.37
S 2	2557	1803	505	0.142	0.267	1.227	1.23	0.28
S 3	6106	3044	907	0.261	0.491	1.48	1.54	0.47
S 4	4961	2810	731	0.264	0.497	1.444	1.44	0.41
S 5	4706	1914	260	0.138	0.261	1.146	1.15	0.29
S 6	3961	2059	831	0.141	0.266	1.291	1.28	0.29
S 7	2635	1608	1199	0.369	0.695	1.651	1.41	0.32
S 8	5735	2259	790	0.248	0.467	1.429	1.55	0.5
S9	6019	2728	1276	0.25	0.471	1.473	1.68	0.53
S 10	3417	2013	1349	0.168	0.316	1.428	1.52	0.36
**Greedy**	**20,832**	**3805**	**617**	**0.803**	**1.512**	**2.03**	**3.9**	**2.61**
S 11	5960	2946	1235	0.213	0.401	1.48	1.69	0.48
S 12	4787	1970	219	0.323	0.609	1.426	1.22	0.35
S 13	4985	2873	685	0.25	0.47	1.436	1.43	0.41
S 14	2636	1448	339	0.109	0.206	1.124	1.05	0.2
S 15	3801	2048	1130	0.26	0.489	1.47	1.5	0.4
S 16	2799	1938	646	0.172	0.325	1.28	1.28	0.3
S 17	4456	2483	634	0.209	0.394	1.344	1.37	0.38
S 18	4288	1823	1186	0.277	0.522	1.49	1.56	0.45
S 19	3979	2359	591	0.181	0.341	1.302	1.35	0.36
S 20	4049	1999	1466	0.226	0.425	1.528	1.64	0.45
**Mean**	**5671.190476**	**2589**	**1025.190476**	**0.247809524**	**0.466714286**	**1.432**	**1.5452381**	**0.48619048**
**S.D ^1^**	**4502.051461**	**1455.031**	**966.4952984**	**0.142480391**	**0.268207968**	**0.192**	**0.56867934**	**0.49363424**

^1^ S.D is the Standard Deviation.

**Table 3:** The intervals of the variable α according to each network.

Network (N)	Interval
$N_{1}$	α ≥ 0.91
Compromised $N_{1}$	0.48 < α ≤ 1.81
$N_{1.2}$	α ≥ 1.5
Compromised $N_{1.2}$	0.093< α ≤ 1.93
$N_{2}$	α ≥ 2
Compromised $N_{2}$	0.28 <α ≤ 2.87
$N_{2.2}$	α ≥ 1.93
Compromised $N_{2.2}$	0.3 < α ≤ 2.93
$N_{3}$	α ≥ 2.17
Compromised $N_{3}$	0.94 < α ≤ 3.53
$N_{3.2}$	α ≥ 1.89
Compromised $N_{3.2}$	0.84 < α ≤ 3.26
$N_{4}$	α ≥ 2.96
Compromised $N_{4.2}$	2.38 ≤ α ≤ 3.37
$N_{4}$	α ≥ 2.56
Compromised $N_{4.2}$	0.52 < α ≤ 1.75

**Table 4:** The thresholds appropriate to each parameter.

Parameter	α Interval	α Value	Threshold
Packets sent (P1)	1.5 ≤ α ≤ 1.75	1.75	$Th_{max}$(P1) = $M_{P1}$+ 1.75 × $\sigma_{P1}$
Packets received (P2)	0.31 ≤ α ≤ 0.54	0.5	$Th_{min}$(P3) = $M_{P2\;}$− 0.5 × $\sigma_{P2}$
Collision (P3)	0.37 ≤ α ≤ 0.42	0.4	$Th_{max}$(P3) = $M_{P3}$+ 0.4 × $\sigma_{P3}$
Transmit power (P4)	1.34 ≤ α ≤ 1.66	1.6	$Th_{max}$(P4) = $M_{P4}$+ 1.6 × $\sigma_{P4}$
Transmit duty cycle (P5)	1.63 ≤ α ≤ 1.66	1.6	$Th_{max}$(P5) = $M_{P5}$+ 1.6 × $\sigma_{P5}$
Power (P6)	1.46 ≤ α ≤ 1.72	1.7	$Th_{max}$(P6) = $M_{P6}$+ 1.7 × $\sigma_{P6}$
Radio on (%) (P7)	1.77 ≤ α ≤ 1.86	1.85	$Th_{max}$(P7) = $M_{P7}$+ 1.85 × $\sigma_{P7}$
Radio Tx (%) (P8)	1.83 ≤ α ≤ 1.84	1.8	$Th_{max}$(P8) = $M_{P8}$+ 1.8 × $\sigma_{P8}$

**Table 5:** The $N_{6}$ network database.

Node	Packets Sent	Collision	Packets Received	Transmit Power	Transmit Duty Cycle	Power	Radio on (%)	Radio Tx (%)
S 1	39,761	29,578	8229	0.279	0.526	1.711	2.68	0.86
S 2	13,825	10,203	2582	0.057	0.107	1.058	1.37	0.37
S 3	4697	2816	384	0.184	0.347	1.228	2.14	0.81
S 4	7299	3952	627	0.099	0.186	1.298	3.08	1.33
S 5	2203	1911	371	0.1	0.189	1.274	1.65	0.46
S 6	4895	2707	853	0.192	0.362	1.464	2.64	1.01
S 7	1599	2251	748	0.124	0.234	1.494	2	0.43
S 8	3280	2507	926	0.347	0.653	1.881	2.75	0.96
S 9	1463	2366	1215	0.113	0.213	1.578	2.31	0.43
S 10	6631	3547	999	0.265	0.498	1.673	288	1.07
**Greedy**	**27,136**	**9775**	**359**	**1.759**	**3.312**	**3.666**	**8**	**5.63**
S 11	2501	2275	396	0.055	0.103	1.189	165	0.41
S 12	3600	2141	643	0.176	0.331	1.444	2.44	0.93
S 13	3788	2341	621	0.075	0.141	1.347	1.88	0.51
S 14	2879	2241	364	0.045	0.085	1.166	1.67	0.46
S 15	5403	3139	1012	0.287	0.541	1.989	2.87	0.96
S 16	10,352	4968	825	0.596	1.123	2.149	3.83	1.87
S 17	4570	3268	878	0.402	0.756	1.812	2.94	1.1
S 18	2506	2408	929	0.203	0.383	1.63	2.32	0.61
S 19	5252	2574	418	0.169	0.318	1.327	2.51	1.13
S 20	745	2065	1028	0.094	0.177	1.523	2.03	0.3
S 21	7448	4251	569	0.122	0.229	1.313	3.17	1.45
S 22	1651	1777	525	0.055	0.104	1.231	1.61	0.35
S 23	2065	2508	811	0.188	0.354	1.572	2.24	0.56
S 24	4896	3458	724	0.097	0.182	1.377	2.82	1.07
S 25	7550	3012	214	1.063	2.002	2.516	2.71	1.42
S 26	1610	2392	1030	0.1	0.188	1.469	2.17	0.42
S 27	3146	2592	1019	0.254	0.479	1.738	2.83	0.9
S 28	8412	4711	931	0.242	0.456	1.679	3.64	1.6
S 29	2227	2458	1051	0.277	0.521	1.791	2.66	0.74
S 30	1166	1582	402	0.058	0.109	1.202	1.37	0.23
**Threshold**	**20,266.8555**	**6680.72717**	**460.387298**	**0.46564168**	**0.87681834**	**2.45005359**	**3.6034103**	**1.74751373**

## Data Availability

Not applicable.
